# Liver indicators affecting the relationship between BMI and hypertension in type 2 diabetes: a mediation analysis

**DOI:** 10.1186/s13098-023-01254-z

**Published:** 2024-01-17

**Authors:** Xuelin Yao, Keyan Hu, Zhen Wang, Fangting Lu, Jie Zhang, Yahu Miao, Qing Feng, Tian Jiang, Yi Zhang, Songtao Tang, Nan Zhang, Fang Dai, Honglin Hu, Qiu Zhang, the China National Diabetic Chronic Complications Study Group

**Affiliations:** Department of Endocrinology, First Affiliated Hospital of Anhui Medical University, 218 Jixi Road, Shushan District, 230032 Hefei China

**Keywords:** Liver indicators, BMI, Hypertension, Type 2 diabetes mellitus, Mediation effect

## Abstract

**Background:**

Body mass index (BMI) is an important risk factor for hypertension in diabetic patients. However, the underlying mechanisms remain poorly understood. Although liver-derived biological intermediates may play irreplaceable roles in the pathophysiology of diabetes, few studies have explored them in the association between BMI and hypertension in diabetes.

**Objective:**

To investigate the role of liver enzymes in mediating the relationship between BIM exposure and hypertension in type 2 diabetes mellitus (T2DM).

**Methods:**

We included a total of 1765 participants from the China National Diabetic Chronic Complications Study Cohort. Associations between liver enzymes and hypertension were estimated using multivariable regression models. The function of liver indicators in the relationship between BMI and hypertension was assessed using mediation analysis. Mediation analysis was conducted, taking into account age, diabetes duration, current smoking, fasting plasma glucose level, glycated hemoglobin, anti-diabetic therapy, and family history of diseases, including diabetes, hypertension, obesity, and hyperlipidemia.

**Results:**

For men, the association of BMI with hypertension was partially mediated by alanine aminotransferase (ALT), with a proportion of mediation was 68.67%, by aspartate aminotransferase (AST) was 27.02%, and by γ-glutamyltransferase (GGT) was 38.58%, by AST/ALT was 63.35%; for women, the proportion mediated by ALT was 36.93%, and by AST was 37.47%, and GGT was 44.60%, and AST/ALT was 43.73% for BMI (all *P* < 0.05).

**Conclusion:**

The effect of BMI on hypertension is partly mediated by liver indicators (ALT, AST, GGT, and AST/ALT) in diabetic patients. Our results may provide opportunities to identify new targets for hypertension interventions.

## Introduction

Diabetes, a metabolic disease characterized by insulin resistance and β-cell dysfunction, is one of the most common and fastest-growing diseases worldwide, projected to affect 693 million adults by 2045 [1]. Type 2 diabetes mellitus (T2DM) accounts for approximately 90% of all diabetes cases and is increasingly recognized as a complex, cardiorenal-metabolic disease entity driven by a chronic positive energy balance [2, 3]. Diabetes and hypertension often occur in tandem, share common risk factors, and lead to increased risks of disability, cancer, and premature death [2, 4–6]. In addition, elevated blood pressure is associated with an increased risk of incident diabetes mellitus [7]. In a longitudinal study, patients with baseline hypertension or even prehypertension had an increased risk of developing diabetes mellitus than normotensive subjects [8]. Furthermore, increasing evidence demonstrates that severe macrovascular and microvascular complications are more likely to occur in diabetic patients with hypertension [9, 10]. However, the risk factors for the development of hypertension and their relative importance in T2DM are not well characterized, limiting the effectiveness of efforts to identify and treat at-risk individuals.

Serum levels of liver enzymes, such as alanine aminotransferase (ALT), aspartate aminotransferase (AST), and to a lesser extent γ-glutamyltransferase (GGT), are routinely measured clinical markers that represent different dimensions of liver dysfunction [11, 12]. ALT, located in the cytosol, and AST, located in the mitochondria, are released from damaged hepatic cells into the blood after hepatocellular injury or death. Elevated liver enzymes have been proposed to play a vital role in the pathophysiology of obesity, hypertension, T2DM, and other metabolic diseases [13, 14]. Increased serum ALT and GGT activities are positively associated with increased risks of developing diabetes and hypertension among adults [15, 16]. Another prospective cohort study showed that GGT could be a potential biomarker among liver enzymes for the early detection of hypertension [17]. Moreover, the AST/ALT ratio (also referred to as the *De Ritis* ratio) has been shown to be a marker for cardiovascular diseases [18].

As the trajectory of hypertension and T2DM are often closely interlinked, this study aims to provide insights into the association between liver function and hypertension among T2DM adults from the China National Diabetic Chronic Complications Study (CDCS) data, comparing them across sex, and body mass index standing. Early detection of the effects of hypertension on diabesity would enable the optimal implementation of effective therapies that prevent macro- and microvascular complications.

## Methods

### Data source and study population

CDCS was conducted by the Chinese Diabetes Society (CDS) and the National Center for Chronic Noncommunicable Disease Control and Prevention of the Chinese Center for Disease Control and Prevention (China CDC), is a large population-based program of multiple chronic complications and co-morbidities of diabetes in China. This program covered all 31 provinces, autonomous regions, and municipalities of mainland China [19]. The current data analysis was based on the prospective cohort study of 1765 Anhui adults with diabetes between March of 2018 and January of 2020. The cohort details have been described elsewhere [20]. Briefly, the participants joined a standard medical examination program provided by the CDCS study. During the physical examinations, blood pressure, and anthropometric measurements (height, weight, and waist circumference) were measured by medical staff according to a standard protocol. Life factors, family history, and medical history of diseases were inquired, and a blood draw for each participant was taken. A more detailed description of the program and examination procedures is reported elsewhere [19].

### Inclusion and exclusion criteria

Enrollment criteria for the target survey population: (1) Household registration location in Anhui Province, diagnosed with diabetes and aged 18–74 years old (time as of December 31, 2018); (2) Permanent residents with an annual residence time of ≥ 6 months; (3) Those who were able to sign an informed consent form, voluntarily participated in the survey of the project, and agreed to complete a blood examination. Individuals who were pregnant or had a debilitating health condition or disease that prevented them from participating, such as being bedridden or mentally disabled, were not eligible to participate.

### Data collection and ethics approval

**Fig. 1:**
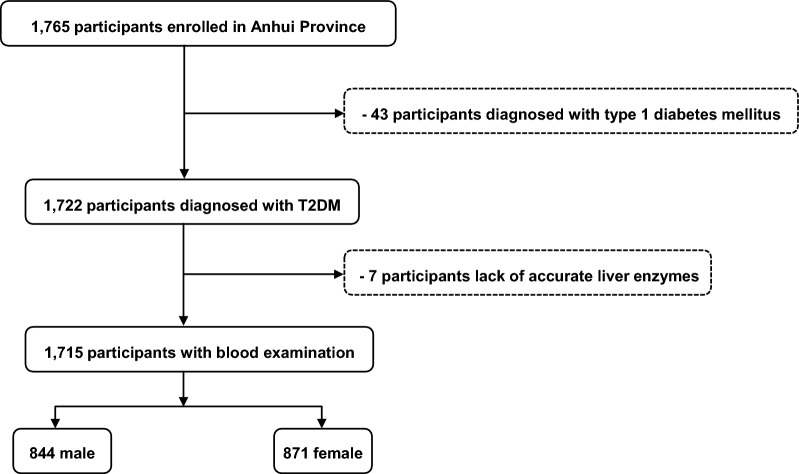
Flow charts for participants’ enrollment

After excluding forty-three respondents with type 1 diabetes mellitus and seven participants with missing information on the blood draw, 1715 participants aged 18–74 years with complete and reliable information (demographic and socioeconomic information, lifestyle factors including smoking, alcohol intake, physical activity, weight measurements, family history of disease and medical history) were included (Fig. 1).

All study procedures were performed by the declaration of Helsinki and relevant guidelines. Institutional review board approval for the study was obtained from the Ethics Committee (Approval No: 2018-010), which was registered in the Chinese Clinical Trial Registry (ChiCTR1800014432). Written informed consent was obtained from each participant before data collection.

### Assessment of variables

Age, gender, life factors (smoking status, alcohol take, physical activity), drug administration, and personal medical history were collected at baseline by trained interviewers using semi-structured questionnaires. Height was measured on a permanently mounted vertical board (TGZ height gauge), according to a standardized protocol. Weight was measured on an electronic scale (TANITA HD-390 body weight scale). The measurement accuracy of height and weight was required to be 0.1 cm and 0.1 kg, respectively. Body mass index (BMI), is a simple calculation based on the ratio of a person’s weight in kilograms divided by height in meters squared. BMI was categorized into normal weight (BMI < 25.0 kg/m^2^), overweight (25.0 ≤ BMI < 30.0 kg/m^2^), and obese (BMI ≥ 30 kg/m^2^) [21]. Waist circumference (WC) was measured in the horizontal plane midway between the lower edge of the costal arch and the upper edge of the iliac crest. The measurement accuracy of WC was required to be 0.1 cm. At both examinations, blood pressure (BP) was measured three times using an automated electronic device (Omron blood pressure monitor) by trained and certified technicians. The onset of the first phase (systolic) and fifth phase (diastolic) Korotkoff sounds were recorded. The mean of the three BP measures was used in the analyses. At the baseline, a 15-mL blood sample was obtained for the determination of routine chemistry between 7:30 and 9:30 AM after an overnight fast for 8–12 h. Mean arterial pressure (MAP) is calculated as diastolic BP + 1/3 × (systolic BP − diastolic BP). MAP combines information from both systolic and diastolic BP into one variable, avoiding collinearity problems that would arise when using the two variables jointly in the statistical model.

Blood samples from all participants were delivered by courier to the local laboratories and Guangzhou KingMed Diagnostics Group Co., Ltd. (Guangzhou, China) in a refrigerator with a temperature range of 2–8 °C for clinical laboratory tests according to a standard protocol. The tests included routine blood examinations, blood lipid examinations, fasting blood glucose measurements, and liver function tests, which measured the fasting plasma glucose level (FPG), glycated hemoglobin (HbA1c), AST, ALT, and GGT levels. Hyperlipidemia was defined by the national guidelines for the management of dyslipidaemias: without lipid-lowering medications, total cholesterol (TC) ≥ 5.20 mmol/L, or triglycerides (TG) ≥ 1.70 mmol/L, or high-density lipoprotein cholesterol (HDL-c) < 1.0 mmol/L, or low-density lipoprotein cholesterol (LDL) ≥ 3.4 mmol/L, or previously diagnosed as hyperlipidemia by a physician [22].

### Outcome and mediator ascertainment

The level of liver indicators (AST, ALT, GGT, and AST/ALT) measured in the baseline survey were estimated as mediators for this study. Hypertension status was assessed using criteria from the national guidelines for primary hypertension prevention and management [23], and the diagnoses included self-reported physician-diagnosed hypertension, blood pressure level ≥ 140/90 mmHg, and use of antihypertensive medication.

### Statistical analysis

Differences in baseline characteristics of the CDCS cohort, overall and stratified by sex and BMI categories were evaluated using one-way analysis of variance (ANOVA) for continuous variables and χ^2^ test for categorical variables. Continuous variables were presented as mean (standard deviation) or mean ± standard deviation and categorical variables were summarized as number (%).

BMI was considered as exposure, liver indicators including ALT, AST, GGT, and AST/ALT as potential mediators, and hypertension as the study outcome. To assess the causal mediation effects, we used the potential outcome framework. Causal mediation analysis [24] based on linear regression with a nonparametric bootstrap was conducted using the R package to obtain the adjusted associations of BMI with liver indicators and the adjusted association of liver indicators with hypertension. We also assessed if the association of BMI with hypertension varied with liver indicators. To facilitate comparison between liver indicators, the *z*-score of BMI and liver indicators were used for data analysis. All analyses were adjusted for age, sex, diabetes duration, current smoking, FPG, HbA1c, anti-diabetic therapy, and family history of diseases, including diabetes, hypertension, obesity, and hyperlipidemia. Given the association of BMI with liver indicators might vary by sex, we assessed whether the association varied by sex from the heterogeneity across strata and the significance of interaction terms in a model.

The value of liver indicators (ALT, AST, GGT, and AST/ALT) were divided into quartiles (Q1: < 25%; Q2: ~ 25%; Q3: ~ 50%; and Q4: ~ 75%), and using the Q1 as the cut-off value. Binary logistic regression analysis was performed to assess the relationship between liver indicators, as either categorical (quartiles) or continuous variable (each absolute 1 SD increase), and the presence of hypertension (yes vs. no). The lowest liver indicator quartiles group was set as the reference, with confounding variables adjusted as mentioned above. All analyses were conducted using the SPSS version 25.0 and R software version 4.2.2. Two-tailed *P* values < 0.05 was considered to indicate significance.

## Results

### Clinical characteristics

Baseline characteristics of the subjects stratified by sex and BMI categories are given in Table 1. A total of 1715 participants (871 females and 844 males) were enrolled in this study, with a mean age of mean ± SD (53.19 ± 9.84) years. The prevalence of hypertension was 50.1% (48.1% among males and 52.0% among females). The mean BMI (kg/m^2^) at baseline was 26.06 ± 3.64 (26.04 ± 3.58 among males and 26.09 ± 3.70 among females); 45.4% of the participants were overweight, and 13.7% obese. The distribution of laboratory parameters and other covariates differed across BMI categories as expected. For all but FPG, HbA1c, LDL, current smoking, family history of hypertension, and physical activity, a significant linear trend was found across BMI categories among overall and different gender (both *P* < 0.05). There was no linear correlation between the age of females with different BMI categories (both *P* > 0.05).

**Table 1:** Participant characteristics of the CDCS cohort, overall and stratified by sex and BMI categories

Characteristics	No. (%) or as mean (SD)											
	Overall			*P* value	Male			*P* value	Female			*P* value
	Normal (*n* = 701)	Overweight (*n* = 779)	Obese (*n* = 235)		Normal (*n* = 337)	Overweight (*n* = 406)	Obese (*n* = 101)		Normal (*n* = 364)	Overweight (*n* = 373)	Obese (*n* = 134)	
Age, years	57.79 (9.40)	57.11 (9.94)	55.63 (10.62)	**0.013**	58.40 (9.52)	56.88 (9.53)	54.25 (11.05)	**0.001**	57.23 (9.27)	57.38 (10.37)	56.66 (10.20)	0.773
WC, cm	81.83 (6.57)	92.75 (5.84)	103.82 (7.10)	**< 0.001**	83.19 (6.27)	94.38 (5.58)	105.51 (7.09)	**< 0.001**	80.57 (6.59)	90.98 (5.59)	102.55 (6.86)	**< 0.001**
SBP, mmHg	143.68 (21.25)	150.17 (20.95)	152.76 (20.01)	**< 0.001**	143.17 (21.68)	148.42 (19.61)	153.88 (20.71)	**< 0.001**	144.15 (20.86)	152.08 (22.18)	151.92 (19.51)	**< 0.001**
DBP, mmHg	80.37 (11.63)	85.15 (11.76)	86.12 (12.06)	**< 0.001**	82.31 (11.78)	87.43 (11.60)	89.81 (12.47)	**< 0.001**	78.57 (11.20)	82.67 (11.45)	83.34 (10.99)	**< 0.001**
MAP, mmHg	101.47 (13.09)	106.82 (12.72)	108.34 (12.85)	**< 0.001**	102.59 (13.50)	107.76 (12.45)	111.17 (13.46)	**< 0.001**	100.43 (12.63)	105.81 (12.96)	106.20 (11.99)	**< 0.001**
FPG, mmol/L	9.55 (3.51)	9.37 (3.00)	9.42 (3.13)	0.574	9.80 (3.49)	9.59 (3.02)	9.29 (3.20)	0.356	9.31 (3.51)	9.13 (2.95)	9.52 (3.09)	0.462
HbA1c, %	7.56 (1.86)	7.58 (1.67)	7.64 (1.63)	0.827	7.67 (1.87)	7.70 (1.66)	7.51 (1.68)	0.637	7.45 (1.85)	7.46 (1.67)	7.73 (1.59)	0.232
TC, mmol/L	5.21 (1.17)	5.20 (1.24)	5.00 (1.06)	**0.048**	5.06 (1.18)	5.20 (1.36)	4.89 (1.14)	0.056	5.35 (1.13)	5.21 (1.09)	5.09 (0.99)	**0.038**
LDL, mmol/L	3.10 (0.98)	3.06 (0.97)	2.98 (0.93)	0.244	3.05 (0.99)	3.02 (0.98)	2.89 (0.92)	0.366	3.15 (0.96)	3.11 (0.95)	3.04 (0.94)	0.549
HDL-c, mmol/L	1.57 (0.47)	1.35 (0.35)	1.27 (0.32)	**< 0.001**	1.53 (0.46)	1.30 (0.37)	1.16 (0.30)	**< 0.001**	1.61 (0.47)	1.41 (0.32)	1.36 (0.32)	**< 0.001**
TG, mmol/L	1.92 (1.86)	2.65 (3.26)	2.52 (2.26)	**< 0.001**	1.78 (1.78)	3.03 (4.24)	2.76 (3.13)	**< 0.001**	2.04 (1.93)	2.34 (1.52)	2.34 (1.24)	0.126
Alb, g/L	51.62 (3.39)	51.37 (3.02)	50.83 (3.13)	**0.005**	51.50 (3.65)	51.82 (3.04)	51.28 (3.41)	0.222	51.73 (3.13)	50.89 (2.93)	50.49 (2.87)	**< 0.001**
GGT, IU/L	31.47 (43.98)	48.30 (53.14)	47.99 (44.36)	**< 0.001**	37.78 (57.27)	59.51 (63.17)	55.85 (41.29)	**< 0.001**	25.62 (24.96)	36.10 (35.71)	42.06 (45.81)	**< 0.001**
ALT, IU/L	21.41 (11.84)	27.86 (19.68)	31.20 (21.32)	**< 0.001**	22.72 (11.91)	30.53 (20.10)	33.13 (17.69)	**< 0.001**	20.20 (11.66)	24.95 (18.57)	29.75 (23.65)	**< 0.001**
AST, IU/L	21.06 (8.08)	24.12 (12.54)	25.67 (14.97)	**< 0.001**	21.61 (8.40)	24.58 (11.85)	24.95 (12.92)	**< 0.001**	20.55 (7.76)	23.63 (13.24)	26.21 (16.38)	**< 0.001**
AST/ALT	1.11 (0.39)	1.00 (0.39)	0.92 (0.32)	**< 0.001**	1.06 (0.40)	0.93 (0.43)	0.83 (0.31)	**< 0.001**	1.15 (0.38)	1.06 (0.33)	0.99 (0.31)	**< 0.001**
Current smoking	134 (19.1)	156 (20.0)	47 (20.0)	0.898	126 (37.4)	151 (37.2)	45 (44.6)	0.369	8 (2.2)	5 (1.3)	2 (1.5)	0.654
Current drinking	180 (25.7)	236 (30.3)	64 (27.2)	0.137	147 (43.6)	203 (50.0)	48 (47.5)	0.222	33 (9.1)	33 (8.8)	16 (11.9)	0.55
Family history of hypertension	424 (60.5)	482 (61.9)	155 (66.0)	0.327	198 (58.8)	251 (61.8)	67 (66.3)	0.361	226 (62.1)	231 (61.9)	88 (65.7)	0.722
Family history of obesity	169 (24.1)	267 (34.3)	117 (49.8)	**< 0.001**	88 (26.1)	134 (33.0)	52 (51.5)	**< 0.001**	81 (22.3)	133 (35.7)	65 (48.5)	**< 0.001**
Family history of CVD	131 (18.7)	147 (18.9)	69 (29.4)	**0.001**	64 (19.0)	77 (17.0)	23 (22.8)	0.664	67 (18.4)	70 (18.8)	46 (34.3)	**< 0.001**
Hypertension	282 (40.2)	428 (54.9)	149 (63.4)	**< 0.001**	127 (37.7)	211 (52.0)	68 (67.3)	**< 0.001**	155 (42.6)	217 (58.2)	81 (60.4)	**< 0.001**
Hyperlipidemia	421 (60.1)	533 (68.4)	168 (71.5)	**< 0.001**	185 (54.9)	273 (67.2)	71 (70.3)	**0.001**	236 (64.8)	260 (69.7)	97 (72.4)	0.186
Lipid lowering medication	83 (11.8)	115 (14.8)	47 (20.0)	**0.007**	32 (9.5)	50 (12.3)	18 (17.8)	0.070	51 (14.0)	65 (17.4)	29 (21.6)	0.111
Antihypertensive drugs	239 (34.1)	370 (47.5)	131 (55.7)	**< 0.001**	103 (30.6)	185 (45.6)	60 (59.4)	**< 0.001**	136 (37.4)	185 (49.6)	71 (53.0)	**< 0.001**
Physical activity	466 (66.5)	515 (66.1)	147 (62.6)	0.528	224 (66.5)	284 (70.0)	61 (60.4)	0.166	242 (66.5)	231 (61.9)	86 (64.2)	0.436

Bold values indicate *P* < 0.05

BMI, body mass index; CVD, cardiovascular disease; WC, waist circumference; SD, standard deviation; FPG, fasting plasma glucose; HbA1c, glycosylated hemoglobin; TG, triglyceride; HDL-c, high-density lipoprotein cholesterol; LDL, low density lipoprotein cholesterol; TC, total cholesterol; SBP, systolic blood pressure; DBP, diastolic blood pressure; FPG, fasting plasma glucose; HbA1c, glycosylated hemoglobin; Alb, albumin; ALT, alanine aminotransferase; AST, aspartate aminotransferase; GGT, γ-glutamyltransferase; AST/ALT ratio, aspartate aminotransferase/alanine aminotransferase

Data are expressed as mean (standard deviation), or number (%)

### Associations of liver indicators with hypertension

Overall, the multivariable-adjusted (age, sex, diabetes duration, current smoking, FPG, HbA1c, anti-diabetic therapy, family history of diseases, including diabetes, hypertension, obesity, hyperlipidemia) odd ratios (ORs) for hypertension across ascending quartiles of ALT were 1.00 (reference), 1.267 (95% confidence interval [CI] 0.956–1.677), 1.505 (95% CI 1.114–2.032), and 1.790 (95% CI 1.341–2.388), respectively. The multivariable-adjusted OR for hypertension in the lowest AST quartile were 1.00 (reference), 1.178 (95% CI 0.886–1.565), 1.233 (95% CI 0.908–1.676), and 1.667 (95% CI 1.256–2.213), respectively. The multivariable-adjusted OR for hypertension in the lowest AST/ALT quartile were 1.00 (reference), 1.128 (95% CI 0.843–1.511), 0.930 (95% CI 0.688–1.258), and 0.647 (95% CI 0.476–0.878), respectively. The multivariable-adjusted OR for hypertension in the lowest GGT quartile were 1.00 (reference), 1.541 (95% CI 1.154–2.057), 1.715 (95% CI 1.262–2.330), and 3.064 (95% CI 2.239–4.194), respectively.

Compared with the lowest liver enzymes quartile, the multivariable-adjusted OR for hypertension in the highest ALT quartile (OR = 1.877, 95% CI 1.224–2.877), highest AST quartile (OR = 1.800, 95% CI 1.189–2.723), and highest AST/ALT quartile (OR = 0.608 95% CI 0.392–0.944), and highest GGT quartile (OR = 3.452, 95%CI 2.129–5.596) remained statistically significant in the subgroup with males. While in the subgroup with females, the multivariable-adjusted OR for hypertension in the highest liver enzymes quartile was slightly and not significant except for GGT.

Although lower levels of AST/ALT were significantly associated with a higher risk of hypertension in the overall group (OR = 0.851, 95% CI 0.763–0.949), and in the subgroup with females (OR = 0.850, 95% CI 0.730–0.990), the association disappeared in males. In addition, despite higher levels of GGT were significantly associated with a higher risk of hypertension in the overall group (OR = 1.234, 95% CI 1.093–1.393), and in the subgroup with male (OR = 1.248, 95% CI 1.048–1.486), the association disappeared in female (Table 2).

**Table 2:** Odd ratios for hypertension by different liver function indicators

	Overall	Female	Male
	(*n* = 1715)	(*n* = 871)	(*n* = 844)
ALT			
Q1 (≤ 15 U/L)	1.00 (ref.)	1.00 (ref.)	1.00 (ref.)
Q2 (15–21 U/L)	1.267 (0.956–1.677)	1.117 (0.766–1.627)	1.462 (0.944–2.263)
Q3 (21–30 U/L)	1.505 (1.114–2.032)	1.239 (0.817–1.879)	1.839 (1.171–2.888)
Q4 (≥ 30 U/L)	1.790 (1.341–2.388)	1.659 (1.097–2.508)	1.877 (1.224–2.877)
* P* for trend	0.001	0.110	0.020
Per SD increase	1.222 (1.098–1.359)	1.240 (1.067–1.441)	1.168 (1.004–1.358)
AST			
Q1 (≤ 16 U/L)	1.00 (ref.)	1.00 (ref.)	1.00 (ref.)
Q2 (16–20 U/L)	1.178 (0.886–1.565)	1.146 (0.776–1.694)	1.202 (0.786–1.839)
Q3 (20–26 U/L)	1.233 (0.908–1.676)	1.210 (0.784–1.868)	1.225 (0.786–1.911)
Q4 (≥ 26 U/L)	1.667 (1.256–2.213)	1.464 (0.983–2.180)	1.800 (1.189–2.723)
* P* for trend	0.004	0.309	0.032
Per SD increase	1.207 (1.085–1.342)	1.203 (1.037–1.395)	1.185 (1.012–1.387)
AST/ALT			
Q1 (≤ 0.78)	1.00 (ref.)	1.00 (ref.)	1.00 (ref.)
Q2 (0.78–0.95)	1.128 (0.843–1.511)	0.972 (0.614–1.539)	1.290 (0.881–1.890)
Q3 (0.95–1.21)	0.930 (0.688–1.258)	0.811 (0.517–1.274)	1.145 (0.753–1.742)
Q4 (≥ 1.21)	0.647 (0.476–0.878)	0.679 (0.433–1.064)	0.608 (0.392–0.944)
* P* for trend	0.002	0.251	0.007
Per SD increase	0.851 (0.763–0.949)	0.850 (0.730–0.990)	0.871 (0.748–1.015)
GGT			
Q1 (≤ 18 U/L)	1.00 (ref.)	1.00 (ref.)	1.00 (ref.)
Q2 (18–27 U/L)	1.541 (1.154–2.057)	1.546 (1.067–2.239)	1.497 (0.921–2.433)
Q3 (27–46 U/L)	1.715 (1.262–2.330)	1.591 (1.040–2.434)	1.723 (1.062–2.796)
Q4 (≥ 46 U/L)	3.064 (2.239–4.194)	2.330 (1.489–3.648)	3.452 (2.129–5.596)
* P* for trend	< 0.001	0.002	< 0.001
Per SD increase	1.234 (1.093–1.393)	1.150 (0.989–1.336)	1.248 (1.048–1.486)

Models were adjusted for age, sex, diabetes duration, current smoking, FPG, HbA1c, anti-diabetic therapy, family history of diseases, including diabetes, hypertension, obesity, hyperlipidemia

Data in the parentheses are 95% uncertainty intervals; OR indicates odds ratio

Q1–Q4 indicate 25th–75th percentile

### Associations of liver indicators with hypertension

**Fig. 2:**
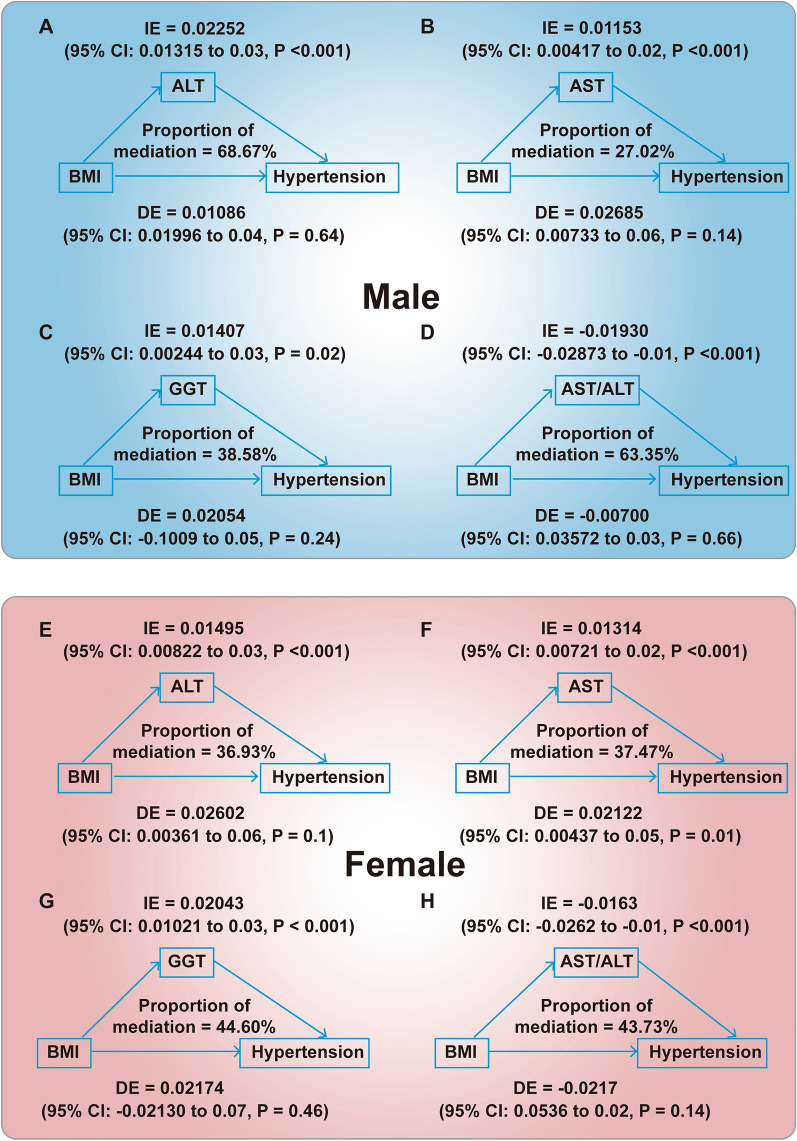
The mediating effect of liver indicators on the BMI associated-hypertension among T2DM adults. The upper (blue) box indicated male patients with T2DM, while the lower (red) box indicated female. **A–D** illustrated how liver indicators, specifically ALT, AST, GGT, and AST/ALT, mediate the link between BMI and hypertension. **E–H** showed the mediating effect of the relationship between BMI and hypertension mediated by liver indicators, including ALT, AST, GGT, AST/ALT, respectively

As the association of hypertension with liver enzymes varied by sex and BMI (*P* values for interaction < 0.001), mediation analysis was conducted in females and males separately. In males, after adjustment for age, diabetes duration, current smoking, FPG, HbA1c, anti-diabetic therapy, and family history of diseases, including diabetes, hypertension, obesity, and hyperlipidemia, the associations of BMI with hypertension were significantly mediated by ALT, AST, GGT and AST/ALT (Fig. 2). In males, the proportion of the associations of BMI with hypertension mediated by ALT was 68.67% (*P* < 0.001) (Fig. 2A). The proportion of the associations of BMI with hypertension mediated by AST was 27.02% (*P* < 0.001) (Fig. 2B). The proportion of the associations of BMI with hypertension mediated by GGT was 38.58% (*P* = 0.02) (Fig. 2C). The proportion of the associations of BMI with hypertension mediated by AST/ALT was 63.35% (*P* < 0.001) (Fig. 2D).

In female, the proportion of the associations of BMI with hypertension mediated by ALT was lower than for male after similar adjustment (36.93%,* P* < 0.001) (Fig. 2E). The proportion of the associations of BMI with hypertension mediated by AST was higher than for male after similar adjustment (37.47%, *P* < 0.001) (Fig. 2F). The proportion of the associations of BMI with hypertension mediated by GGT was higher than for males after similar adjustment (44.60%, *P* < 0.001) (Fig. 2G). The proportion of the associations of BMI with hypertension mediated by AST/ALT was lower than for males after similar adjustment (43.73%, *P* < 0.001) (Fig. 2H).

## Discussion

The present study aimed to determine the degree to which liver indicators, such as ALT, AST, GGT, and AST/ALT, mediate the known effect of BMI on hypertension in adults with T2DM. We found that ALT most significantly mediated the effects of BMI on hypertension, followed by AST/ALT. The regulatory effect of ALT and AST/ALT was greater in males than females. Recent studies have confirmed the impact of BMI on hypertension or liver enzymes on hypertension [17]. However, here we first assessed the extent to which these effects of BMI on hypertension are mediated by liver indicators based on a large diabetes cohort study and proposed a further target for hypertension prevention or intervention.

Data from previous studies have reported that high BMI relates to increased DNA methylation age in a tissue-specific manner [25]. The authors report an unexpectedly strong correlation between high BMI and the epigenetic age of liver tissue and also suggest that the increased age of liver tissue in obese individuals may provide insights into liver-related comorbidities of obesity, such as insulin resistance, diabetes, and hepatocellular carcinoma [25]. Recent epidemiologic studies also showed an association between BMI and liver function with hypertension in type 2 diabetic outpatients but did not formally test mediation [26, 27]. We found that liver indicators (including ALT, AST, GGT, and AST/ALT) partially mediated the impact of BMI on hypertension, which may be more relevant in the population than at the individual level. Given that this mediating effect is partial, BMI may also have a direct impact on hypertension or an effect through other possible mediators.

In our study, males had stronger BMI-ALT and BMI-AST/ALT associations than females (all *P* < 0.01). Females had a stronger BMI-AST and BMI-GGT association than males (all *P* < 0.01). The possible explanation for this finding is the complex interplay of female and male sex hormones, such as estrogens. The effect of estrogens on liver enzymes and hypertension received recently more attention, and it was shown that genetic mechanisms contribute to body fat distribution in women and men [28, 29]. Women store fat preferentially in subcutaneous adipose tissue, men store fat preferentially in the visceral and white adipose tissue. Aromatase activity in the white adipose tissue increases estrogen levels in elderly or obese men above those in women [30]. These estrogens are important to hypertension and liver function disorders in men. Furthermore, at the same BMI, visceral adipose percentage may be higher in men than in women, which may lead to a higher risk for poor liver function [31]. Therefore, due to pronounced differences in the regulation of fatty acid metabolism between males and females, statistical analysis needs to be conducted separately by gender. On the other hand, Asian women carry greater abdominal and visceral fat than their Caucasian counterparts with similar total adiposity, which may result in a higher metabolic risk for obesity-related diseases, such as diabetes and hypertension [32, 33]. As with any causal inference method, mediation analysis requires assumptions to be made about the causality of the effects in the mediation model. Specifically, it is assumed that changes in BMI cause changes in liver enzymes and that changes in liver enzymes cause changes in hypertension. In addition, statistically, liver enzymes were considered as mediators rather than confounders because the causal association of BMI with liver enzymes including ALT, AST, and GGT [34], and the causal association of ALT and GGT activities with hypertension is supported by a recent study [15]. The statistical method used in our study thus enabled us to provide a more straightforward and robust estimate of the mediation effect.

To date, several studies have examined relationships between liver dysfunction and the risk of hypertension [16, 17, 35], however, the results regarding AST/ALT remain inconclusive. Increasing evidence has suggested that AST/ALT is associated with an increased risk of cardiovascular diseases, DM, peripheral arterial disease, and nonalcoholic fatty liver disease. Yet current information regarding the association between AST/ALT and hypertension is relatively scarce. A cross-sectional study of 14,220 Chinese hypertensive patients, and indicated that increased AST/ALT ratio levels were predictive of all-cause and cardiovascular mortality among Chinese hypertensive patients [36].

There are several limitations of the current study that should be clarified. First, although the novelty analytic methodological framework employed in the present study has been used previously [14, 37], it has not been applied to BMI and liver indicators among diabetic patients. Second, our models were based on measures of BMI and liver indicators from a single point in time which might not reflect the risk associated with lifetime exposure to higher BMI or changes in liver enzymes over time. Measurement errors could attenuate the estimates of the mediation. Third, this study adopted a single mediator model. Multiple mediators may affect one another, and these mediators may act as confounders of the effects of other mediators [38]. Finally, since liver diseases such as fatty liver and cirrhosis are of concern, we intend to conduct more advanced professional surveys at a later date, which would provide extensive theoretical support for our findings.

## Conclusions

The effect of BMI on hypertension was partly mediated by liver indicators. This study re-emphasizes the importance of liver function for hypertension prevention and intervention and offers opportunities to identify new targets for hypertension interventions among diabetic patients.

## Data Availability

The original contributions presented and analyzed during the current study are available from the corresponding author for reasonable use.

## Abbreviations

T2DM: Type 2 diabetes mellitus
CVD: Cardiovascular disease
FPG: Fasting plasma glucose
HbA1c: Glycosylated hemoglobin
OR: Odds ratio
CI: Confidence interval
CDCS: The China National Diabetic Chronic Complications Study
WC: Waist circumference
BMI: Body mass index
SBP: Systolic blood pressure
DBP: Diastolic blood pressure
TC: Total cholesterol
TG: Triglycerides
HDL-c: High-density lipoprotein cholesterol
LDL: Low-density lipoprotein cholesterol
ALT: Alanine aminotransferas
AST: Aspartate aminotransferas
GGT: γ-Glutamyltransferase
